# Integrating machine learning algorithms and multiple immunohistochemistry validation to unveil novel diagnostic markers based on costimulatory molecules for predicting immune microenvironment status in triple-negative breast cancer

**DOI:** 10.3389/fimmu.2024.1424259

**Published:** 2024-06-28

**Authors:** Chao Zhang, Wenyu Zhai, Yuyu Ma, Minqing Wu, Qiaoting Cai, Jiajia Huang, Zhihuan Zhou, Fangfang Duan

**Affiliations:** State Key Laboratory of Oncology in South China, Guangdong Provincial Clinical Research Center for Cancer, Sun Yat-Sen University Cancer Center, Guangzhou, Guangdong, China

**Keywords:** triple-negative breast cancer, costimulatory molecules, diagnostic biomarker, tumor immune microenvironment, machine learning algorithm

## Abstract

**Introduction:**

Costimulatory molecules are putative novel targets or potential additions to current available immunotherapy, but their expression patterns and clinical value in triple-negative breast cancer (TNBC) are to be clarified.

**Methods:**

The gene expression profiles datasets of TNBC patients were obtained from The Cancer Genome Atlas and the Gene Expression Omnibus databases. Diagnostic biomarkers for stratifying individualized tumor immune microenvironment (TIME) were identified using the Least Absolute Shrinkage and Selection Operator (LASSO) and Support Vector Machine-Recursive Feature Elimination (SVM-RFE) algorithms. Additionally, we explored their associations with response to immunotherapy via the multiplex immunohistochemistry (mIHC).

**Results:**

A total of 60 costimulatory molecule genes (CMGs) were obtained, and we determined two different TIME subclasses (“hot” and “cold”) through the K-means clustering method. The “hot” tumors presented a higher infiltration of activated immune cells, i.e., CD4 memory-activated T cells, resting NK cells, M1 macrophages, and CD8 T cells, thereby enriched in the B cell and T cell receptor signaling pathways. LASSO and SVM-RFE algorithms identified three CMGs (CD86, TNFRSF17 and TNFRSF1B) as diagnostic biomarkers. Following, a novel diagnostic nomogram was constructed for predicting individualized TIME status and was validated with good predictive accuracy in TCGA, GSE76250 and GSE58812 databases. Further mIHC conformed that TNBC patients with high CD86, TNFRSF17 and TNFRSF1B levels tended to respond to immunotherapy.

**Conclusion:**

This study supplemented evidence about the value of CMGs in TNBC. In addition, CD86, TNFRSF17 and TNFRSF1B were found as potential biomarkers, significantly promoting TNBC patient selection for immunotherapeutic guidance.

## 1 Introduction

Triple-negative breast cancer (TNBC) accounts for nearly 15% of all breast cancers (1). Due to its paucity of definitive targets and the intrinsic aggressiveness, most TNBC-related deaths occur, and it remains a grave life-threatening disease among women worldwide (2–5). In view of lacking estrogen receptor (ER), progesterone receptor (PR), and human epidermal growth factor receptor 2 (HER2), TNBC can’t benefit from endocrine and targeted treatments, and depend on traditional chemotherapy with little clinical benefits realized, a median overall survival (OS) about 12 to 18 months (6–9).

In recent years, immune-checkpoint inhibitors (ICIs) have revolutionized the therapeutic landscape of multiple tumors, such as nasopharyngeal carcinoma, melanoma, and lung cancer, wherein, ICIs generated durable responses, resulted in substantial survival progress, and have been recommended as a part of standardized treatments (10). Compared with other breast cancer subtypes, TNBC exhibits stronger immunogenicity, abundant tumor-infiltrating lymphocytes (TILs), higher programmed cell death ligand 1 (PD-L1) expression and tumor mutation burden (TMB) (2, 11, 12), numerous explorations on adding ICIs to the therapeutic arsenal of TNBC have acquired inspiring feedback (13–15). While survival benefits derived from ICIs in TNBC are relatively minimal as compared to other tumors (2, 16, 17). Therefore, identifying and developing optimal biomarkers have become a hot area. Currently, PD-L1 and TMB are most used predictors for patient selection in clinical practice, but absence of standardized criteria for the methodology and expression cutoff values leads to their inconsistency predictive value in different clinical trials and therapeutic regimes (16, 18, 19). Although a possible association between higher TILs and improved pCR rates from an immunotherapeutic perspective has been examined, it is confined to the early stage of TNBC (20). Hence, novel predictors of immunotherapeutic response are necessary and meaningful to appropriately select ideal patients who can benefit from ICIs with the aim to design individualized strategies.

The tumor immune microenvironment (TIME), composed of various immune cells, stromal cells, mesenchymal cells, cytokines, and chemokines, plays a crucial role in the processes of tumor initiation, progress, development, and metastasis (21). A deep parsing of the diversity and complexity of TIME is valuable for improving anti-tumor immune responses and patient stratification according to their unique TIME classes and subclasses, thereby greatly improving therapeutic benefits from ICIs and unraveling novel targets (22). Increasing evidence suggests that features associated with the “hot” tumor, including abundant TILs in TIME, markers related to T cell activation, as well as signatures for adhesion, are potential factors for predicting responses to ICIs (23). Costimulatory molecules, comprising the B7-CD28 family with 13 molecules and the tumor necrosis factor (TNF) family with 48 molecules, are vital for the differentiation, proliferation, maturation, survival, activation, and functions of immune cells. The former includes the most common PD-1 and PD-L1 axis of ICIs, the latter includes 19 members belong to the TNF ligand superfamily (TNFSF) and 29 members to the TNF receptor superfamily (TNFRSF) (22, 24, 25). Moreover, they are putative novel targets or potential additions to current available immunotherapeutic strategies (26, 27). The predictive model based on costimulatory molecule genes (CMGs) have been explored in lung adenocarcinoma (28), while their functions and clinic value in TNBC are little illustrated.

Herein, we aimed to systematically dissect the expression pattern and clinical value of costimulatory molecules in TNBC. Using the transcriptional profiles of TNBC patients from The Cancer Genome Atlas (TCGA) and the Gene Expression Omnibus (GEO) databases, we stratified patients into two different TIME status (“cold” and “hot”) through the K-means clustering method and compared their difference in immune cell infiltrations via the CIBERSORT algorithm (29). Subsequently, Least Absolute Shrinkage and Selection Operator (LASSO) (30) and Support Vector Machine-Recursive Feature Elimination (SVM-RFE) (31) were utilized to identify diagnostic markers from CMGs. Following, a diagnostic signature for stratifying individualized TIME status of TNBC patients was stablished, and its predictive performance was further validated. Moreover, we conducted a small-sample exploratory analysis via the multiplex immunohistochemistry (mIHC) to explore the correlation between the expression level of identified CMGs biomarkers and response to immunotherapy.

## 2 Materials and methods

### 2.1 Data acquisition and preparation

We downloaded the gene expression profile datasets of TNBC patients from TCGA (https://tcga-data.nci.nih.gov/tcga/) and GEO databases (https://www.ncbi.nlm.nih.gov/geo/) (GSE76250 and GSE58812 datasets) using the “GEO query” package (32). All microarray datasets were standardized via the “SVA” and “limma” R packages. TCGA, GSE76250, and GSE58812 datasets consisted of 168, 165, and 107 tumor samples, respectively. A total of 60 CMGs, including 13 members of B7-CD28 family and 47 members of TNF family, were obtained from a previously published study (**Supplementary Table 1**) (28).

### 2.2 Patient-clustering based on CMGs

To investigate the potential value and functions of abovementioned 60 CMGs in the TIME of TNBC, we classified patients into different clusters after the k-means machine learning algorithm, an unsupervised consensus clustering method, using the “Cluster” package. First, we determined the corresponding optimal cluster numbers in three datasets via the “factoextra” package. After k-means clustering, we performed the principal component analysis (PCA) with the “factoextra” package. Next, we utilized the “ESTIMATE” package (33) to calculate and compare the tumor purity, immune, and stromal scores among different clusters in TCGA, GSE76250, and GSE58812 datasets. TNBC patients in three datasets were further stratified into the “hot” and “cold” tumor groups according to their immune and stromal scores.

### 2.3 Estimation of the immune cell infiltration landscape in TIME

The standardized gene expression profiles of TNBC patients in TCGA, GSE76250, and GSED58812 datasets were performed by the CIBERSORT algorithm with perm set to 1000 to analyze the characteristics of 22 immune cells infiltration (29) between patients belonging to the “hot” and “cold” tumor groups.

### 2.4 Functional annotation and pathway enrichment analyses

Gene set enrichment analysis (GSEA, https://www.gsea-msigdb.org/gsea/index.jsp) was performed for patients in “hot” vs “cold” tumors through the Java GSEA (version4.0.1) and the Kyoto Encyclopedia of Genes and Genomes (KEGG) pathway in C2 and Gene Ontology (GO) terms in C5 to evaluate potential functional pathways and biological mechanisms enriched in patients (34). False discovery rate (FDR) < 0.25 and normalized *P* < 0.05 were set as cutoffs to obtain significant enrichment.

### 2.5 Screening and identification of the diagnostic CMGs biomarkers

In the TCGA and GSE76250, to avoid possible influence of multicollinearity, we firstly conducted the LASSO logistic regression analysis through the “glmnet” package to screen out biomarkers from above all 60 CMGs at the optimal value of log lambda with the smallest classification error (30). Besides, the SVM-RFE machine learning algorithm based on the support vector machine was utilized to identify the most valuable biomarkers from all 60 CMGs by subtracting the feature vector determined using SVM with the “e1071” and “caret” R packages (31). Next, we merged identified CMGs from above-mentioned machine learning algorithms via the “scMerge” package to further narrow the number of markers. These overlapped CMGs markers were finally input for the logistics regression analysis to identify the final diagnostic biomarkers.

### 2.6 Conduction and validation of the diagnostic nomogram based on CMGs biomarkers

Based on abovementioned final CMGs biomarkers, a diagnostic signature for individualized TIME status was constructed and visually presented as nomogram via the “rms” R package. Then, we evaluated and validated the predictive accuracy and clinical value of the CMG-based nomogram using the receiver operating characteristic (ROC) curves, calibration curves, and decision curve analysis (DCA) in both training and validation datasets.

### 2.7 Tissue multiplex immunohistochemistry

We stained TNBC samples using the multiplex fluorescence immunohistochemical kit, PDOne four-color TSA-RM-275 (20 T) (cat 10001100020 Panovue, Beijing, China) according to the manual provided. Paraffin-embedded samples were sequentially incubated with primary antibodies and horseradish peroxidase (HRP)-conjugated secondary antibodies. Then, we performed the tyrosine signal amplification (TSA) to label antigens, after each TSA labeling step, we removed the primary and secondary antibodies through a microwave treatment for heat-induced antigen retrieval. After the sample was eluted, the next antigen was labeled, and this procedure was repeated for all four antigen markers. Anti-CD86 (E2G8P, dilution 1:200, Rabbit, Cell Signaling Technology, Danvers, MA, USA), TNFRSF17 (ab245940, dilution 1:100, Rabbit, Abcam, Cambridge, UK) and TNFRSF1B (28746–1-AP, dilution 1:200, Proteintech, Rosemont, USA) were utilized as primary antibodies. The dyes Opal520, Opal570, Opal650 and 4′-6′-diamidino-2-phenylindole (DAPI, Sigma-Aldrich) were utilized for staining. We scanned TNBC samples and obtained their fluorescence images at ×20 magnification with a PanoVIEW VS200 slide scanner (Panovue, Beijing, China) and an Olympus 20× lens. Image recognition and analysis were performed with QuPath image analysis software (Version 0.3.0, Queen’s University of Belfast, Northern Ireland, UK). The images were quantized into data by tissue segmentation and cell segmentation using the positive threshold settings and phenotypic recognition. By means of an R script (Version 4.0.1), we assumed the quantitative data and basic data such as the positive cell number, positive staining rate and density for subsequent analysis.

### 2.8 Statistical analysis

In current study, the expression levels of CMGs in TNBC patients were presented as raw and standardized data. We conducted this study in two phases. During the first phase, we classified TNBC patients into the “hot” and “cold” tumors according to their immune and stromal scores. During the second phase, TCGA dataset was used as the training cohort, TCGA and the GSE76250 datasets were utilized as internal validation cohorts, and the GSE58812 dataset was as external validation cohort. A diagnostic signature based on identified CMGs biomarkers for predicting individualized TIME subclasses in TNBC was constructed and validated.

All statistical analyses herein were performed using the R software (version 4.0.1, Vanderbilt University, Nashville, TN). The Mann-Whitney U test and Kruskal-Wallis H test were utilized to compare the immune score, stromal score, and tumor purity among different clusters. The unpaired Student’s t test was performed to compare differences in responses to immunotherapy among patients with TNBC. A *P*-value <0.05 was considered statistically significant unless specified.

## 3 Results

### 3.1 Data extraction and processing

**Figure 1:**
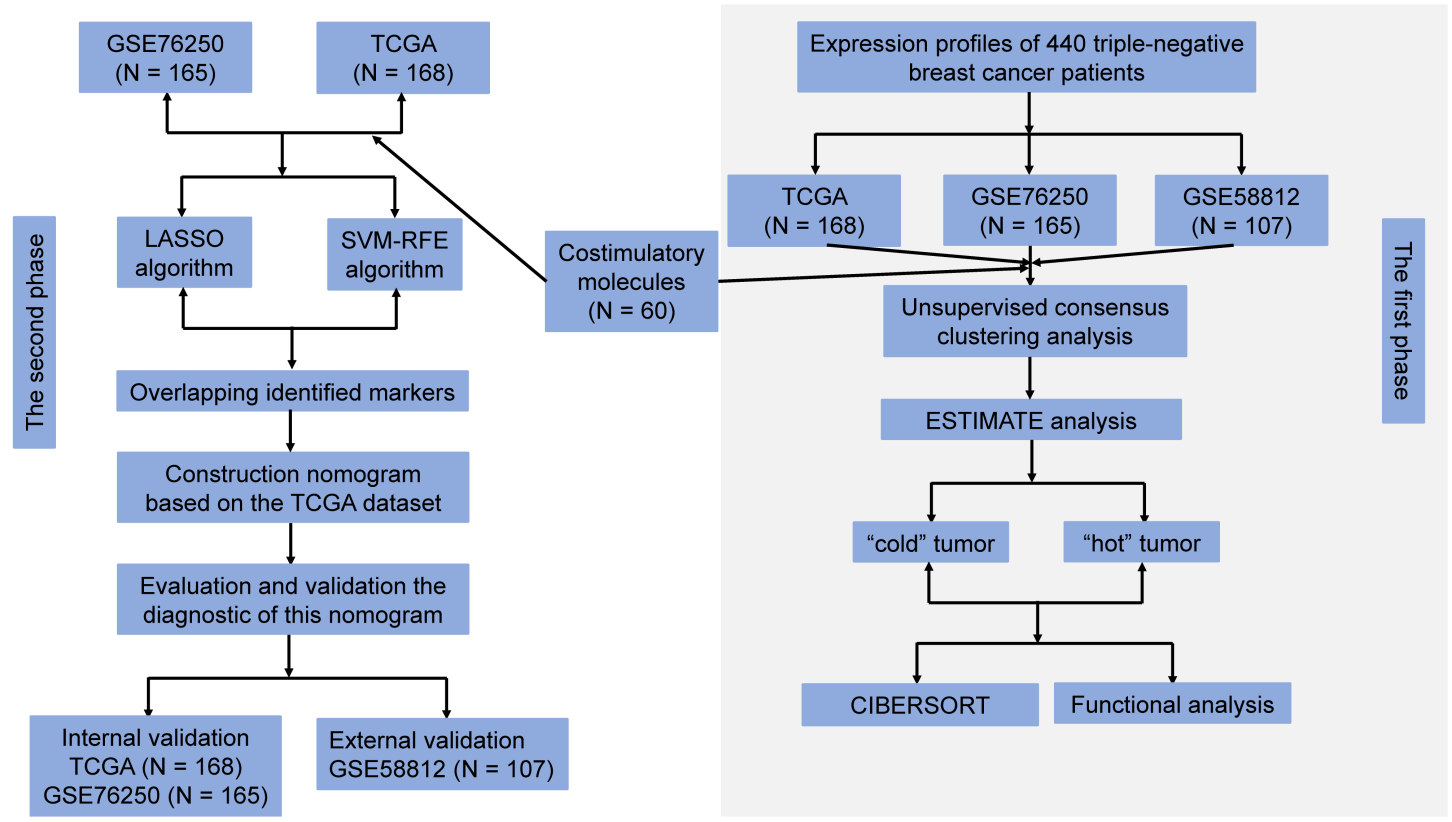
Flowchart of the study design.

The flow chart for the study design is presented in **Figure 1**. All microarray matrixes of three publicly available datasets were annotated, following which, TCGA dataset consisted of 168 TNBC samples (46999 genes), GSE76250 and GSE 58812 datasets included 165 TNBC samples (30906 genes) and 107 TNBC samples (20161 genes), respectively. Next, 60 CMGs were overlapped with TCGA dataset, except for the TNFRSF6B gene due to its low expression, a total of 59 CMGs were identified. Similarly, 60 CMGs were merged with GEO datasets, wherein, only 57 CMGs in the GSE76250 dataset and 58 CMGs in the GSE58812 dataset were eligible. Then, we standardized the expression levels of CMGs in three datasets using the “SVA” and “limma” R packages. Finally, a total of 56 CMGs were used for subsequent analysis.

### 3.2 Patient-clustering based on CMGs

**Figure 2:**
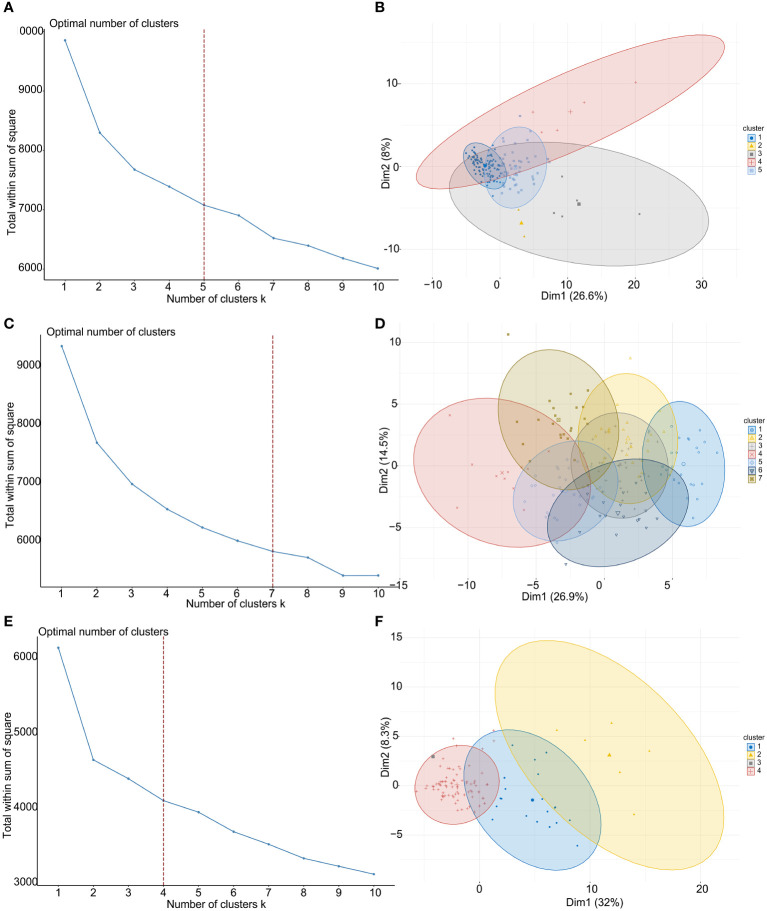
TNBC patient-clustering based on costimulatory molecule genes (CMGs). **(A)** The curve of the total within the sum of squared error curve for the corresponding cluster number *k* in TCGA dataset; **(B)** The principal component analysis (PCA) plot of clustered patients in TCGA dataset; **(C)** The curve of the total within the sum of squared error curve for the corresponding cluster number *k* in GSE76250 dataset; **(D)** The PCA plot of clustered patients in GSE76250 dataset; **(E)** The curve of the total within the sum of squared error curve for the corresponding cluster number *k* in GSE58812 dataset; **(F)** The PCA plot of clustered patients in GSE58812 dataset.

To explore the clinical value and functions of above CMGs in TNBC, an unsupervised consensus clustering analysis was performed to stratify patients. **Figures 2A, C, E** show the curves of the total within the sum of squared error for the corresponding cluster numbers of *k*. These suggested that a *k* value of 5, 7, and 4 were the most optimal in TCGA, GSE76250, and GSE58812 datasets, respectively. The PCA was performed to evaluate the credibility of these cluster numbers, and it demarcated five clusters at *k* = 5 in TCGA dataset (**Figures 2B**). Similarly, patients were distinguished at k = 7 in the GSE76250 dataset (**Figure 2D**), and *k* = 4 in the GSE58812 dataset (**Figures 2F**).

Next, we used the “ESTIMATE” R package (33) to estimate the tumor purity, and to calculate the percentages of stromal and immune cells infiltrations in TIME of TNBC patients based on their CMGs expression profiles. It showed that the tumor purity among patient clusters was significantly different for TCGA, GSE76250 and GSE58812 datasets (**Supplementary Figure 1**). Besides, significant differences among multiple clusters for tumor stroma and immune scores in TCGA (**Supplementary Figures 2A, B**), GSE76250 (**Supplementary Figures 2C, D**), and GSE58812 (**Supplementary 2E, F**) datasets were also observed. Accordingly, we classified the TNBC patients in cluster 1 of TCGA dataset into the “cold” tumor group, while those in clusters 2 to 5 were in the “hot” tumor group. In the GSE76250 dataset, we categorized patients in clusters 4 and 5 as the “hot” tumor group and patients in other clusters formed the “cold” tumor group. In the GSE58812 dataset, TNBC patients in clusters 1 and 2 were divided into the “hot” tumor, while the remaining patients were in the “cold” tumor group.

**Figure 3:**
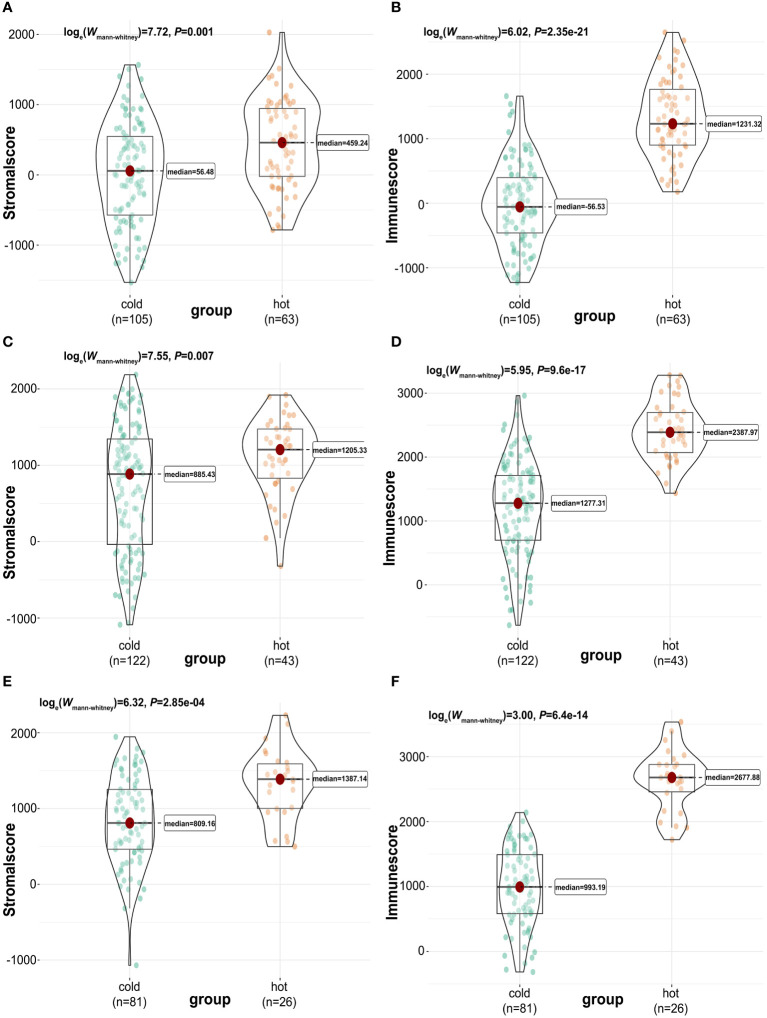
Calculation and comparison of tumor-stromal and immune scores between different TIME subclasses in TNBC, where red represents patients in the “hot” tumor and green shows patients in the “cold” tumor. The comparison of stromal scores **(A)** and immune scores **(B)** between “hot” (clusters 2 to 5) and “cold” (cluster 1) tumor groups in TCGA dataset; The comparison of stromal scores **(C)** and immune scores **(D)** between “hot” (clusters 4 and 5) and “cold” (clusters 1,2,3,6 and 7) tumor groups in GSE76250 dataset; The comparison of stromal scores **(E)** and immune scores **(F)** between “hot” (clusters 1 and 2) and “cold” (clusters 3 and 4) tumor groups in GSE58812 dataset.

Next, we used the “ESTIMATE” R package (33) again to calculate and compare the tumor purity, stromal, and immune cells infiltrations between “cold” and “hot” tumors. There were significant differences in the stromal and immune cell types among TCGA (**Figures 3A, B**), GSE76250 (**Figures 3C, D**), and GSE58812 datasets (**Figures 3E, F**). A significantly higher tumor purity in the “cold” tumor relative to the “hot” tumor in TCGA, GSE76250, and GSE58812 datasets (**Supplementary Figure 3**) was observed.

### 3.3 Estimation of the immune cell infiltration landscape in TIME

**Figure 4:**
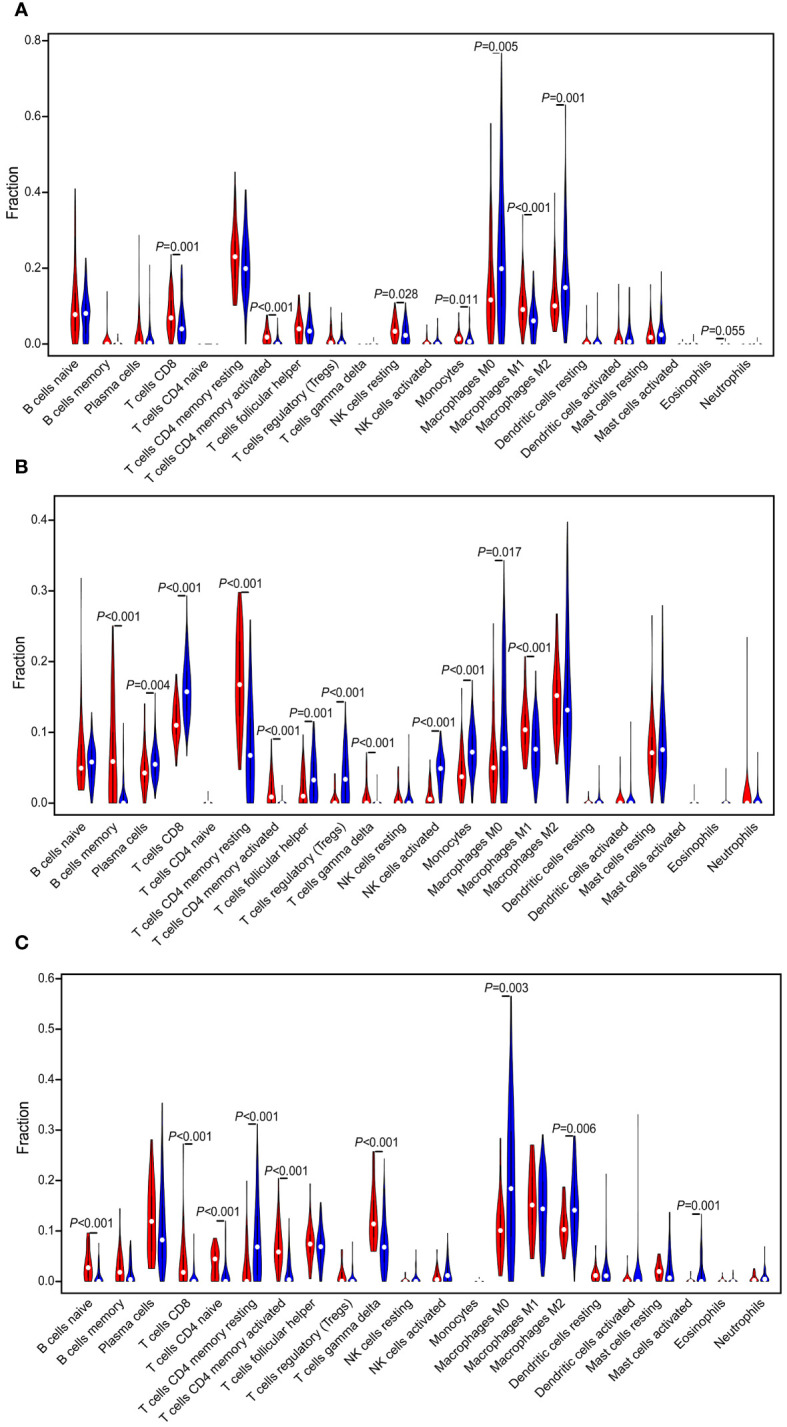
Evaluation and visualization of 22 immune cell type infiltration landscape between different TIME status. The violin plot depicts infiltration disparities among immune cell types between the “hot” tumor (red) and the “cold” tumor (blue) in TCGA **(A)**, GSE76250 **(B)**, and GSE58812 datasets **(C)**.

The landscape of 22 immune cell type infiltrations in TNBC tissues were estimated based on the gene expression profiles via the CIBERSORT algorithm (29), and we also evaluated the correlation between the immune cells in the TIME of TNBC patients. In TCGA dataset, the distribution of immune cells between “cold” and “hot” tumors were significantly different, including CD8 T cells (*P* = 0.001), CD4 memory activated T cells (*P* < 0.001), resting NK cells (*P* = 0.028), monocytes (*P* = 0.011), M0 macrophages (*P* = 0.005), M1 macrophages (*P* < 0.001), and M2 macrophages (*P* = 0.001). Among them, the proportion of M0 and M2 macrophages were higher in the “cold” tumor (*P* < 0.05), while that of others was higher in the “hot” tumor (*P* < 0.05) (**Figure 4A**). Significant differences in the proportion of immune cells between the “cold” and “hot” tumors were also found in GSE76250 dataset, namely, memory B cells (*P* < 0.001), CD8 T cells (*P* < 0.001), resting CD4 memory T cells (*P* < 0.001), CD4 memory activated T cells (*P* < 0.001), follicular helper T cells (*P* = 0.001), regulatory T cells (Tregs) (*P* < 0.001), activated NK cells (*P* = 0.028), monocytes (*P* = 0.011), M0 macrophages (*P* = 0.017), and M1 macrophages (*P* < 0.001). Among them, the distributions of memory B cells, resting CD4 memory T cells, CD4 memory activated T cells, and M1 macrophages were higher, while CD8 T cells, follicular helper T cells, Tregs, activated NK cells, monocytes and M0 macrophages were lower in the “hot” tumor (*P* < 0.05) (**Figure 4B**). In GSE58812 dataset, multiple immune cell types infiltrating the “cold” and “hot” tumors showed significantly different distributions, including the naïve B cells (*P* < 0.001), CD8 T cells (*P* < 0.001), CD4 naïve T cells (*P* < 0.001), resting CD4 memory T cells (*P* < 0.001), CD4 memory activated T cells (*P* < 0.001), M0 macrophages (*P* = 0.003), and M2 macrophages (*P* = 0.006). Among them, the proportions of naïve B cells, CD8 T cells, CD4 naïve T cells, and CD4 memory-activated T cells were higher in the “hot” tumor (*P* < 0.05) (**Figure 4C**).

Additionally, the correlation matrix for the 22 immune cells in TNBC tissues was constructed (**Supplementary Figure 4**). For example, a positive correlation between CD4 memory-activated T cells and M1 macrophages (Cor = 0.37), and CD4 memory-activated T cells and CD8 T cells (Cor = 0.31) was observed in TCGA dataset (**Supplementary Figure 4A**). Likewise, a positive correlation between CD8 T cells and activated NK cells (Cor = 0.66) in GSE76250 dataset (**Supplementary Figure 4B**), a positive correlation between CD4 memory activated T cells and M1 macrophages (Cor = 0.31) in the GSE58812 dataset (**Supplementary Figure 4C**) were observed.

### 3.4 Functional annotation and pathway enrichment analyses

**Figure 5:**
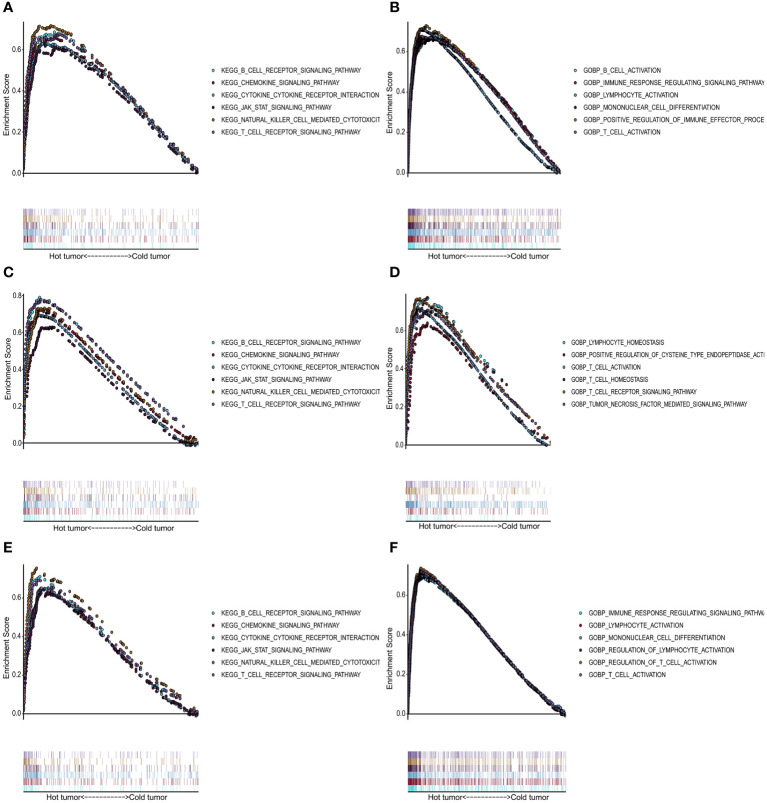
Functional analysis for the “hot” tumor and the “cold” tumor based on costimulatory molecule genes (CMGs). **(A, C, E)** The Kyoto Encyclopedia of Genes and Genomes (KEGG) enrichment analysis in TCGA, GSE76250, and GSE58812 datasets, respectively; **(B, D, F)** Gene Ontology (GO) analysis for biological processes in TCGA, GSE76250, and GSE58812 datasets, respectively.

We performed GO and KEGG enrichment analyses for the “cold” and “hot” tumors to reveal potential functions and pathways. It demonstrated that “hot” tumor group was enriched in the chemokine signaling pathway, cytokine-cytokine receptor signaling pathway, JAK-STAT signaling pathway, nature killer cell-mediated signaling pathway, B cell receptor, and T cell receptor signaling pathway in TCGA (**Figure 5A**), GSE76250 (**Figure 5C**), and GSE58812 datasets (**Figure 5E**). As for the biological processes (BP), the “hot” tumor was mainly associated with the T cells activation and the regulation of immune responses in all three datasets (**Figures 5B, D, F**).

### 3.5 Screening and identification of the diagnostic CMGs biomarkers

**Figure 6:**
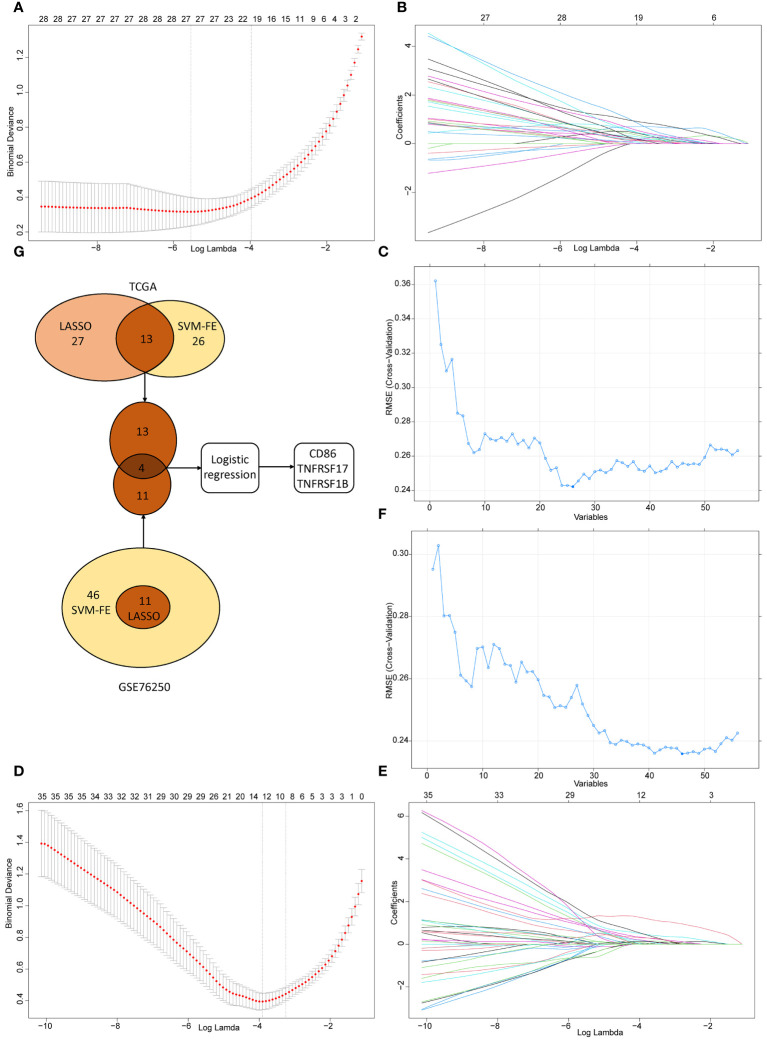
The selection of diagnostic biomarkers from candidate costimulatory molecule genes (CMGs). The lower abscissa is the log lambda value, while the upper abscissa is the number of CMGs with non-zero coefficient; the vertical axis represents the Least Absolute Shrinkage and Selection Operator (LASSO) coefficient of CMGs, and each curve shows the variation trajectory of the coefficients of each gene. **(A)** Determination of the number of CMGs with non-zero coefficients at the optimal value -5.830026 of log lambda in TCGA dataset; **(B)** LASSO coefficient profiles of 27 candidate CMGs after the 10-fold cross-validation in TCGA dataset; **(C)** Support Vector Machine-Recursive Feature Elimination (SVM-RFE) method to identify markers in TCGA dataset; **(D)** Definition of the number of CMGs with non-zero coefficients at the optimal value -3.836916 of log lambda in GSE76250 dataset; **(E)** LASSO coefficient profiles of 35 candidate CMGs after the 10-fold cross-validation in GSE76250 dataset; **(F)** SVM-RFE method to identify markers in GSE76250 dataset; **(G)** Venn diagram presents the overlapping diagnostic markers identified by LASSO and SVM-REF algorithms.

In TCGA dataset, we performed the LASSO logistic regression analysis and screened out 27 CMGs from 56 candidates, having zero coefficients at the optimal value -5.830026 of log lambda (**Figures 6A, B**). 26 CMGs from 56 candidates were recognized as diagnostic biomarkers based on the result of SVM-RFE algorithm (**Figure 6C**). Diagnostic biomarkers identified using above two algorithms were overlapped and 13 CMGs remained to be biomarkers (**Figure 6G**). Similarly, in GSE76250 dataset, 11 CMGs and 46 CMGs from 56 candidates were identified as putative diagnostic biomarkers via the LASSO (a value -3.836916 of the optimal log lambda) (**Figures 6D, E**) and SVM-RFE (**Figures 6F**) machine learning algorithms. Among these, 11 CMGs were overlapping (**Figure 6G**). Next, we merged candidate CMGs identified from above two datasets and performed the logistic regression analysis to further narrow the number of diagnostic biomarkers, three CMGs (CD86, TNFRSF17, and TNFRSF1B) were determined as final diagnostic biomarkers (**Figure 6G**). All CMGs analyzed in this phase were listed in **Supplementary Table 2**.

### 3.6 Construction and validation of the diagnostic nomogram based on CMGs

**Figure 7:**
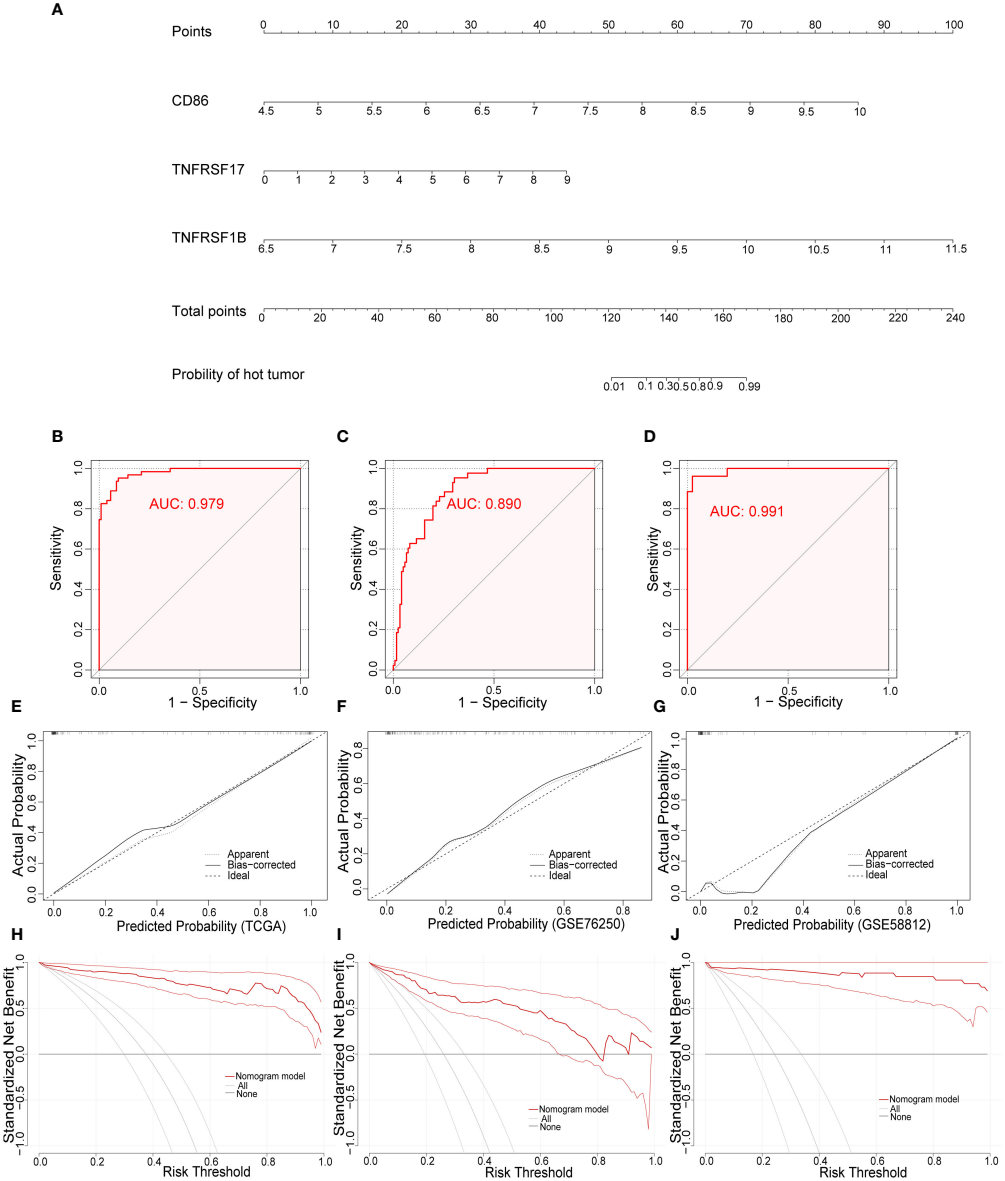
Development and validation of the diagnostic nomogram. **(A)** A nomogram for diagnosing individualized tumor immune environment subclass. The receiver operating characteristic (ROC) curve of the diagnostic efficacy verification in TCGA **(B)**, GSE76250 **(C)**, and GSE58812 datasets **(D)**; The calibration plots of the diagnostic accuracy validation in three datasets **(E–G)**; The decision curve analysis (DCA) of the clinical value for therapeutic guidance in TCGA **(H)**, GSE76250 **(I)**, and GSE58812 datasets **(J)**.

To develop a practical tool for individually predicting TIME subclass in patients with TNBC, we constructed the diagnostic nomogram incorporating above three final CMGs biomarkers, including CD86, TNFRSF17, and TNFRSF1B, based on findings in TCGA (**Figure 7A**). Each biomarker could be scored in points line according to its expression, and after summation, every TNBC patient could have a total score, based on which, the probability of “hot” tumor could be predicted by locating the total score on the probability of the hot tumor scale. For example, TNBC patients with high expression of above three CMGs were more likely to be recognized as “hot” tumor. We further assessed the diagnostic efficiency of this nomogram in three datasets by calculating the area under the ROC curve (AUC), which suggested a satisfactory diagnostic accuracy in TCGA (**Figure 7B**), GSE76250 (**Figure 7C**), and GSE58812 datasets (**Figure 7D**). Moreover, a favorable agreement between actual and predicted probability through the diagnostic nomogram in all three datasets was observed via the calibration curves (**Figures 7E–G**). Additionally, the DCA results demonstrated a good clinical value of this diagnostic nomogram in predicting TIME status (**Figures 7H–J**).

### 3.7 Expression of CMGs markers positively related to efficacy of immunotherapy

**Figure 8:**
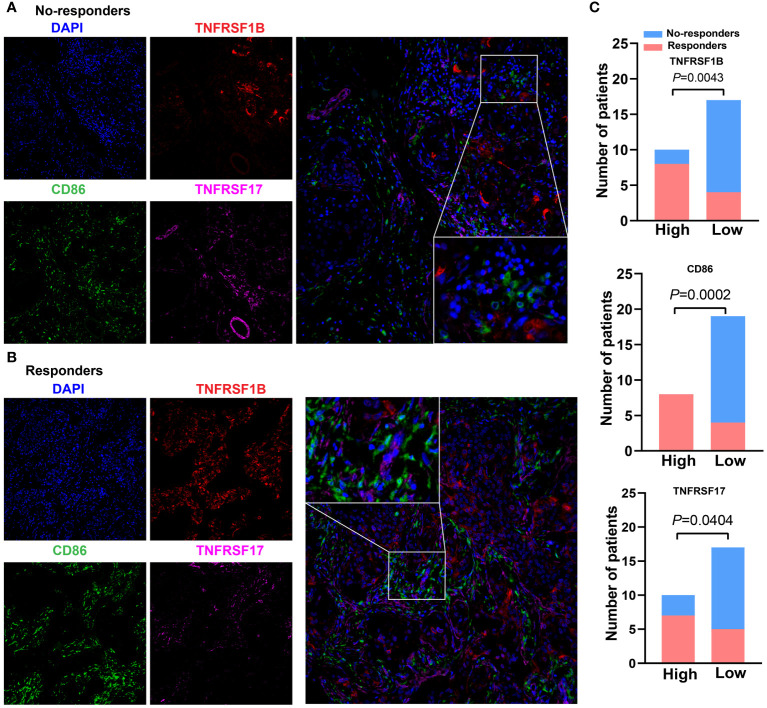
Expression of CMGs biomarkers are positively related to the efficacy of immunotherapy in TNBC. Representative multiplex immunofluorescence images demonstrating the protein expression of CD86 (blue), TNFRSF1B (red) and TNFRSF17 (purple) in samples from nonresponders **(A)** and responders **(B)**; **(C)** Correlation analysis showed that TNFRSF1B, CD86 and TNFRSF17 were significantly associated with the immune response.

To further verify the associations between the expression level of these three CMGs biomarkers in tumor tissues of TNBC patients receiving immunotherapy and patients’ response to treatment, we collected 27 patients’ paraffin-embedded samples and performed the mIHC assays. **Figures 8A**, **B** showed the representative mIHC images of DAPI and three CMGs biomarkers in tumor tissues of responders and no-responders, i.e., DAPI (blue), CD86 (green), TNFRSF17 (purple) and TNFRSF1B (red). Further statistical analysis demonstrated that there were significant associations between high expression of CMGs biomarkers and patients’ positive response to immunotherapy (**Figure 8C**).

## 4 Discussion

In recent years, the therapeutic landscape of TNBC patients has broadened owing to the rapid development of immunotherapy, but only a few patients benefit from ICIs treatment unlike the excellent therapeutic responses achieved in other tumors (16). Effective biomarkers for predicting responses to ICIs in TNBC are lacking in the clinical setting (19). Increasing evidence demonstrated that understanding the unique subsets of personalized TIME is meaningful for identifying novel therapeutic targets and guiding immunotherapeutic strategies (21). In current study, we creatively classified TNBC patients into the “hot” and “cold” tumors according to their TIME clusters determined by CMGs. Further, we executed two machine learning algorithms and identified three CMGs (CD86, TNFRSF17, and TNFRSF1B) as diagnostic biomarkers, based on which, a diagnostic nomogram for predicting TIME subclasses in TNBC in TCGA dataset was constructed, which presented satisfactory predictive accuracy and good clinical value in both the training and validation datasets. Moreover, exploratory analysis in a real-world clinic cohort of patients with TNBC via mIHC also revealed an apparently positive association between the expression level of these three CMGs biomarkers with responses to immunotherapy. This suggest that these CMGs biomarkers might be promising tool for TNBC patients’ stratification to immunotherapeutic guidance.

The failure of current immunotherapy targeting CTLA-4 or PD-1 may be caused by intratumor T cell exhaustion (2), therefore increasing interests revolve around costimulatory molecules residing in the TIME in TNBC tumors. The costimulatory molecules mainly include the B7-CD28 family and the TNF family of proteins (24, 25). Their expressions on tumor cell and lymphocyte surfaces play important roles in regulating the anti-tumor immune responses (22). The immune surveillance function of the immune system in the human body helps to distinguish malignant from normal cells and initiates subsequent attacks. During this process, the naïve T cells should be activated through two indispensable signals, one of which is the costimulatory signal (35), so the activation of T cells could be blocked without costimulatory signals (28). Generally, malignant cells deliver incorrect messages to T cells and prevent the recognition of costimulatory signals by altering their structures and expressions in TIME, and further inducing an immunosuppressive TIME, thereby helping tumor cells to evade immune-induced elimination (36). A single-cell RNA profile analysis of B cells in breast cancer showed that tumors elicited immune-suppressive B cells owing to their failure in extracting costimulatory signals from them, which facilitated further breast tumor cell evasion of immune surveillance (37). ICIs, by blocking the PD1-PD-L1 and the CD86/CTLA4 axes, prevent tumor cells from releasing wrong messages to T cells, thereby restoring tumor-induced immuno-deficiency in TIME (38). While except for common realized PD1/PD-L1 and CD86/CTLA4 axes, many costimulatory members are still poorly understood. To explore the clinical value of costimulatory molecules in TNBC, we obtained 56 CMGs from public databases in this study. Utilizing the unsupervised consensus clustering algorithm, we clustered patients into two different TIME subclasses, namely the “cold” tumor and the “hot” tumor. TIME, composed of several immune cells, carcinoma-associated fibroblasts, stromal cells, and tumor endothelial stromal cells, plays a crucial role in multiple biological processes, including tumor initiation, angiogenesis, and immune regulation (21, 22). Classifying the immune contexts within a TIME represents the first level of cognizing immunological composition and status (activated or suppressed), their influence on survival outcomes and responses possibilities to anti-tumor treatment. Moreover, TIME classifications might also promote to understand its principle of affecting the establishment and maintenance of specific immunological compositions (21).

Tumors were generally classified as “cold” with immune deficiency if their TIME population included immune cells but lacked activation, which could promote metastasis and disease progression as adaptive immunity could not recognize extrinsic antigens or malignancies (39). Whereas, a high expression of activation biomarkers, such as PD-L1, on activated immune cells or tumor cells within a tumor, are the key characteristics of an immunological “hot” tumor, which is likely to initiate anti-tumor immune responses to attack tumors (21). Herein, we simultaneously examined the immune cells infiltrations in the “hot” and “cold” tumors to elucidate the differences in their TIME. The findings demonstrated that immunosuppressive cells, such as M0 and M2 macrophages, had significant infiltration in the “cold” tumor. Typically, M1 macrophages secret cytokines to activate T cells and exert antitumoral effects, while M2 macrophages are pro-tumorigenic factors via angiogenesis and the chemotaxis of Tregs (40). Therefore, the presence of poor immunogenic TIME in patients with “cold” tumors was reasonably speculated. The “hot” tumor exhibited significantly higher infiltrations of various activated immune cells, including CD4 memory activated T cells, resting NK cells, M1 macrophages, and CD8 T cells, which demonstrated that TNBC patients with “hot” tumors had an immuno-active TIME. Similarly, our functional enrichment analysis presented that B cell and T cell receptor-signaling pathways were significantly enriched in “hot” tumors. Significant associations with the activation of T cells and the regulation of immune responses were found in “hot” tumors. Complex interactions between immunosuppressive cells cooperate to suppress the anti-tumor immune responses and promote disease progression. Hence, our findings might provide a reference for guiding combinatorial immunotherapy strategies. For example, a patient with “hot” TNBC tumors might respond to a single ICI, resulting in the intensification of the preexisting anti-tumor benefits and further prolonging survival. However, in TNBC patients with “cold” tumors, a single-agent ICI might not be sufficient. Thus, radiotherapy, chemotherapy, or novel therapeutic strategies, such as inducible T cells co-stimulator (ICOS) agonist, NCT03829501, transforming growth factor-beta (TGF-β) inhibitor, NCT04429542, along with ICIs might unleash the silent anti-tumor immunity and further generate promising clinical prognosis by transforming the “cold” tumor into a “hot” tumor (2, 16, 41).

Different predictive models focus on the TIME and immune landscape for TNBC clinical prognosis and therapeutic sensitivity, but most of them only emphasized clusters and characteristics of intratumoral immunes cells, mRNA panels, and/or protein signatures, and their clinical applications remain to be clarified (42–45). Given the current unsatisfactory immunotherapeutic benefits in patients with TNBC, patient selection using reliable biomarkers for predicting responses is necessary (2). PD-L1, TILs, and TMB are commonly used to guide treatment, but they were subjected to inconsistent results and low predictive accuracy in different clinical trials (16, 18–20). Herein, we highlighted the comprehensive landscape and diagnostic value of CMGs, for the exploration of novel biomarkers. Moreover, traditional prognostic signatures were established through an individual-based model, which required the recognition of survival event information a priory, i.e., it was “supervised”. In current study, we executed the unsupervised consensus clustering algorithm based on expression profiles of CMGs to evaluate the characteristic subclasses of TIME, which could maximize the homogeneity of immune composition within the same cluster and the heterogeneity among different clusters (46). In addition, we identified candidate CMGs rigorously by integrating LASSO regression analysis with the SVM-RFE machine learning algorithm to reinforce the statistical power of the results.

Subsequently, we identified three CMGs (CD86, TNFRSF17, and TNFRSF1B) as diagnostic markers by numerous bioinformatics. CD86, also termed as B70 (B7–2), exerts a suppressive role through CTLA-4 on T cells activation. Thus, its competitive stimulation signal by binding to CD28 is crucial in immune responses, survival of T lymphocytes, and generation of cytokines (47). In the stimulatory status, CD86 can up-regulate its expression via antigen-presenting cells (APCs), and further combine with CD28 delivering stimulation signals to promote anti-tumor immune and enhance activating T cells (48, 49). TNFRSF17, a transmembrane glycoprotein, also known as B cell maturation antigen, is preferentially expressed by mature B lymphocytes and critically regulate B cell proliferation and survival, as well as maturation and differentiation into plasma cells. Previous studies indicated that TNFRSF17 has a dispensable role in overall B cells homeostasis and is an important surface protein supporting the survival of multiple myeloma cells (50, 51). Preclinical models found that the overexpression and activation of TNFRSF17 was associated with multiple myeloma, supporting its potential utility as a therapeutic target. And significant clinical responses in patients with refractory multiple myeloma who failed at least three prior treatments had been achieved by the anti-TNFRSF17 antibody-drug conjugate (52). TNFRSF1B or TNFR2, a member of the TNF receptor superfamily, is expressed by T cells, deliver activating signals, which are largely dependent on antigen recognition and participate in activation, clonal expansion, and differentiation of T cells. Accumulating evidence in recent years indicates that costimulatory signals via TNFR2 plays indispensable roles in protective immunity, inflammatory, autoimmune diseases, and tumor immunotherapy (53).

Based on above three diagnostic markers, we developed a diagnostic nomogram for TNBC patients, which showed that patients with high expressions of CD86, TNFRSF17, and TNFRSF1B had a high probability of “hot” tumor. Besides, satisfactory predictive performance of this nomogram was validated in three independent datasets, including the TCGA, GSE76250, and GSE58812. The “hot” TNBC tumors were mainly related to the BPs of T cell activation and immune response regulation, which implied that patients with “hot” tumors might likely respond to immunotherapy. In consistent with above presumption, the mIHC results showed that responders to immunotherapy were significantly associated with high expressions of CD86, TNFRSF17 and TNFRSF1B in TNBC patients. Hence, our diagnostic nomogram has the potential to aid identifying ideal TNBC patients who may benefit from ICIs, thereby providing immunotherapeutic guidance.

There were some limitations in this study. First, although we included three different independent datasets in current study, it is a retrospective analysis and all data were obtained from public databases, so practical bias might be unavoidable. Second, the underlying mechanism of these three diagnostic markers (TNFRSF17, CD86 and TNFRSF1B) remain poorly understood. Third, we performed this research by means of bioinformatics, further experimental validation of their predictive ability and clinical value are needed in future. Finally, although we constructed our diagnostic model based on CMGs from TCGA data consisting of samples from the United States and validated it using GEO datasets comprising of populations from France and China, prospective studies in different populations are warranted to further validate these results.

## 5 Conclusions

In summary, we comprehensively parse the expression patterns and clinical value of costimulatory molecules in TNBC patients and further clustered patients into two TIME subclasses (“hot” and “cold”) for patients’ stratification. In addition, we identified three CMGs (CD86, TNFRSF17 and TNFRSF1B) as putative diagnostic markers, based on which, a novel diagnostic nomogram for predicting TIME status were constructed and validated with good predictive accuracy and clinical value. This may provide a new insight into the value of CMGs in stratifying TIME status of patients with TNBC, which might serve as a tool to identify ideal candidates and tailor rational immunotherapeutic strategies for TNBC patients.

## Data availability statement

The datasets presented in this study can be found in online repositories. The names of the repository/repositories and accession number(s) can be found in the article/**Supplementary Material**.

## Ethics statement

The studies involving humans were approved by the Ethics Committee of Sun Yat-Sen University Cancer Center. The studies were conducted in accordance with the local legislation and institutional requirements. The ethics committee/institutional review board waived the requirement of written informed consent for participation from the participants or the participants’ legal guardians/next of kin because Written informed consent from all participants was waived due to its retrospective nature.

## Author contributions

CZ: Writing – review & editing, Writing – original draft, Formal analysis, Data curation. WZ: Methodology, Writing – review & editing, Writing – original draft, Formal analysis. YM: Data curation, Writing – review & editing, Writing – original draft. MW: Writing – review & editing, Data curation. QC: Writing – review & editing, Data curation. JH: Project administration, Funding acquisition, Writing – review & editing. ZZ: Writing – original draft, Conceptualization, Writing – review & editing, Project administration. FD: Methodology, Formal analysis, Data curation, Writing – review & editing, Writing – original draft, Conceptualization.

## Supplementary material

Supplementary Figure 1 Comparison of tumor purity among different TNBC patient clusters. The comparison of tumor purity in TCGA **(A)**; GSE76250 **(B)**; and GSE58812 datasets **(C)**.

Supplementary Figure 2 Comparison of tumor-stromal and immune scores among different TNBC patient clusters. The comparison of stromal scores **(A)** and immune scores **(B)** among different clusters in TCGA dataset; The comparison of stromal scores **(C)** and immune scores **(D)** among different clusters in GSE76250 dataset; The comparison of stromal scores **(E)** and immune scores **(F)** among different clusters in GSE58812 dataset.

Supplementary Figure 3 Comparison of tumor purity between different immune environment subclasses. Significant differences in tumor purity between the “cold” and the “hot” tumor groups in TCGA **(A)**; GSE76250 **(B)**; and GSE58812 datasets **(C)**.

Supplementary Figure 4 The correlation heat map shows the correlation of 22 immune cells between two TIME subclasses in TCGA **(A)**, GSE76250 **(B)**, and GSE58812 datasets **(C)**. The number within colored squares represents the strength of the correlation; the larger is the number, the stronger is the correlation. Blue represents a negative correlation and red represents a positive correlation.
