# Supplementary figures and images for: Integrating machine learning algorithms and multiple immunohistochemistry validation to unveil novel diagnostic markers based on costimulatory molecules for predicting immune microenvironment status in triple-negative breast cancer
Source: Front Immunol. 2024 Jun 28;15:1424259. doi: 10.3389/fimmu.2024.1424259 (PMC11239375; doi:10.3389/fimmu.2024.1424259)

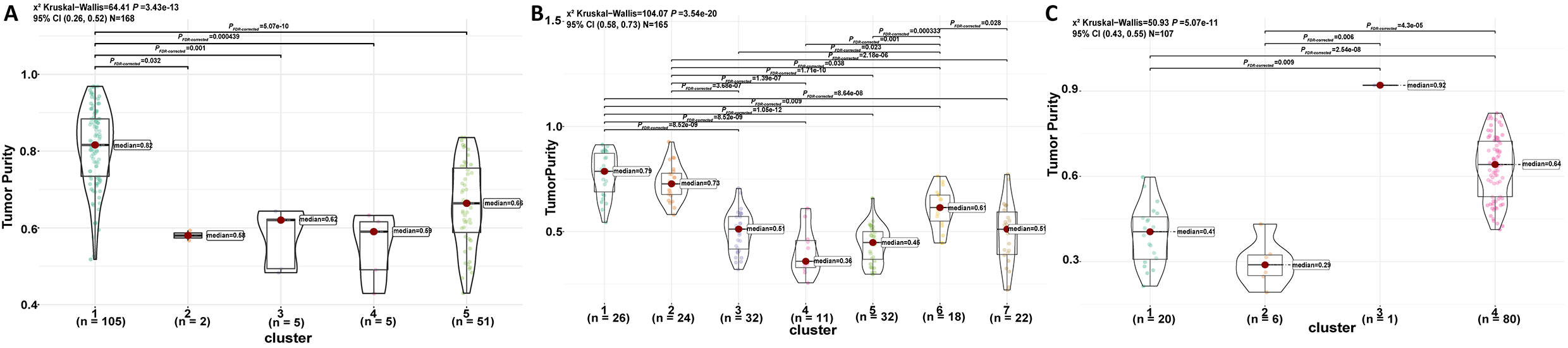

Supplement: Supplementary Figure 1 — Comparison of tumor purity among different TNBC patient clusters. The comparison of tumor purity in TCGA (A); GSE76250 (B); and GSE58812 datasets (C). [file Image_1.tif]

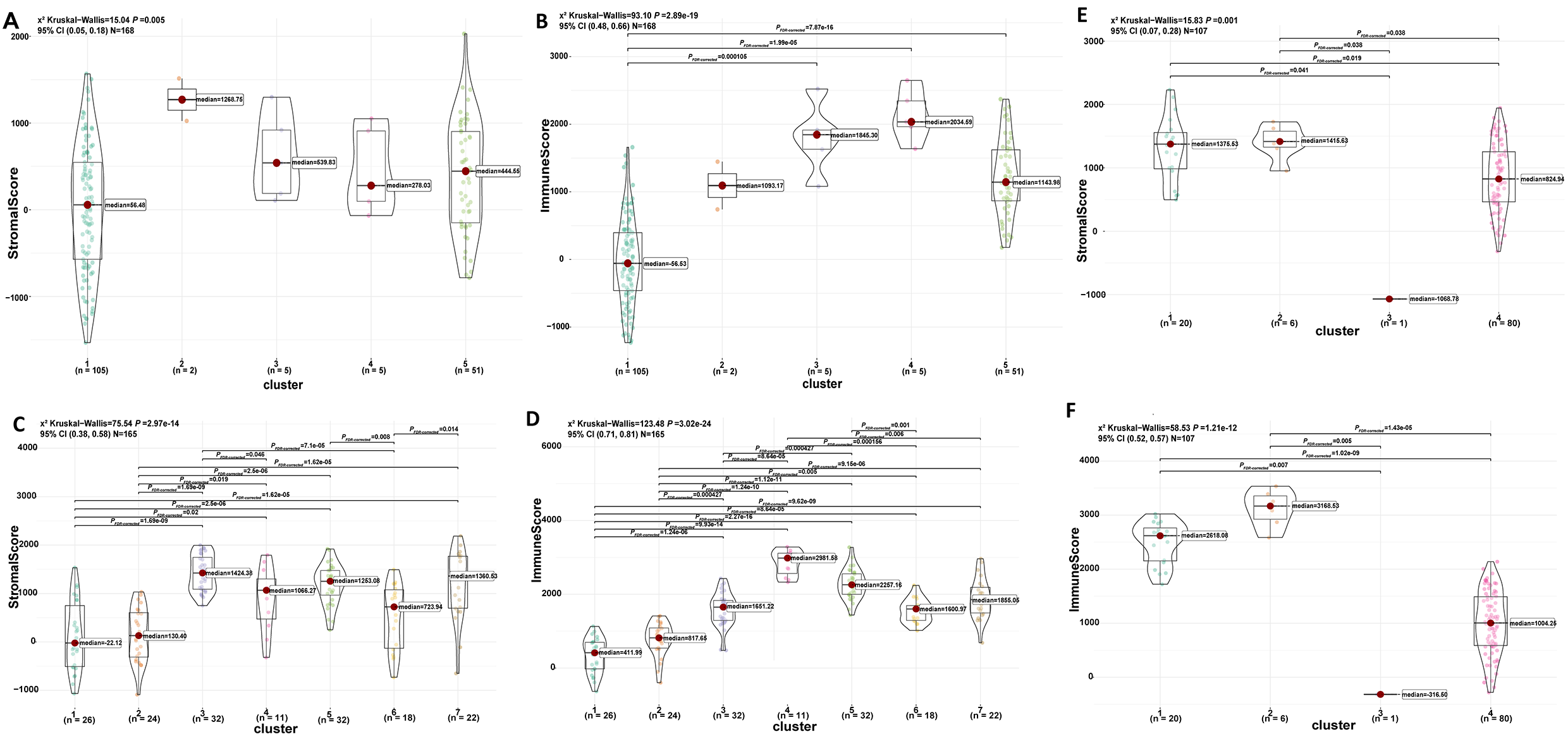

Supplement: Supplementary Figure 2 — Comparison of tumor-stromal and immune scores among different TNBC patient clusters. The comparison of stromal scores (A) and immune scores (B) among different clusters in TCGA dataset; The comparison of stromal scores (C) and immune scores (D) among different clusters in GSE76250 dataset; The comparison of stromal scores (E) and immune scores (F) among different clusters in GSE58812 dataset. [file Image_2.tif]

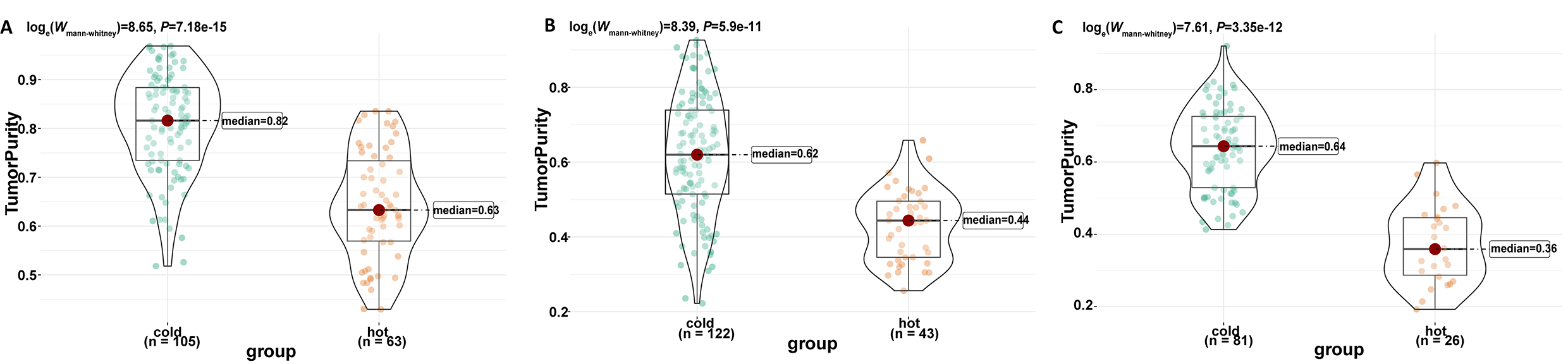

Supplement: Supplementary Figure 3 — Comparison of tumor purity between different immune environment subclasses. Significant differences in tumor purity between the “cold” and the “hot” tumor groups in TCGA (A); GSE76250 (B); and GSE58812 datasets (C). [file Image_3.tif]

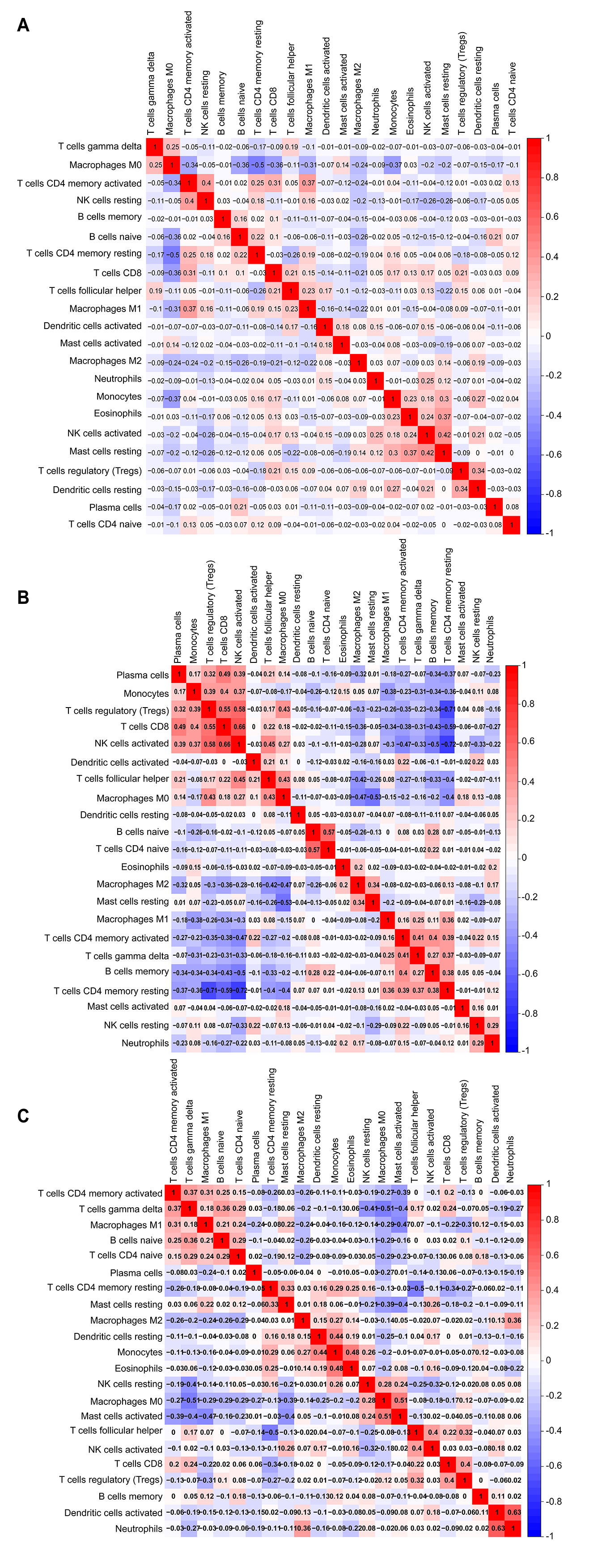

Supplement: Supplementary Figure 4 — The correlation heat map shows the correlation of 22 immune cells between two TIME subclasses in TCGA (A), GSE76250 (B), and GSE58812 datasets (C). The number within colored squares represents the strength of the correlation; the larger is the number, the stronger is the correlation. Blue represents a negative correlation and red represents a positive correlation. [file Image_4.tif]
