# Supplementary material for: Integrating machine learning algorithms and multiple immunohistochemistry validation to unveil novel diagnostic markers based on costimulatory molecules for predicting immune microenvironment status in triple-negative breast cancer
Source: Front Immunol. 2024 Jun 28;15:1424259. doi: 10.3389/fimmu.2024.1424259 (PMC11239375; doi:10.3389/fimmu.2024.1424259)
Supplement: Supplementary file 6 [file Table_2.docx]

**Supplementary Table 2:** Selection of costimulatory molecules genes using the Least

Absolute Shrinkage and Selection Operator (LASSO) and the Support Vector Machine-

Recursive Feature Elimination (SVM-RFE) in TCGA and GSE76250 datasets.

| **TCGA** | | **GSE76250** | |
| --- | --- | --- | --- |
| **LASSO** | **SVM-RFE** | **LASSO** | **SVM-RFE** |
| CD276 | CD27 | CD40LG | CD27 |
| CD40 | CD274 | CD86 | CD274 |
| CD70 | CD28 | CTLA4 | CD276 |
| CD86 | CD40 | LTB | CD28 |
| EDA | CD40LG | TNFRSF13C | CD40 |
| FAS | CD70 | TNFRSF17 | CD40LG |
| FASLG | CD80 | TNFRSF1B | CD70 |
| ICOSLG | CD86 | TNFSF10 | CD80 |
| PDCD1 | CTLA4 | TNFSF18 | CD86 |
| PDCD1LG2 | FASLG | TNFSF4 | CTLA4 |
| RELT | ICOS | TNFSF8 | FAS |
| TNFRSF10B | LTA |  | FASLG |
| TNFRSF11A | LTB |  | ICOS |
| TNFRSF12A | PDCD1 |  | ICOSLG |
| TNFRSF14 | PDCD1LG2 |  | LTA |
| TNFRSF17 | TMIGD2 |  | LTB |
| TNFRSF18 | TNFRSF13B |  | LTBR |
| TNFRSF19 | TNFRSF14 |  | PDCD1 |
| TNFRSF1B | TNFRSF17 |  | PDCD1LG2 |
| TNFRSF4 | TNFRSF1B |  | RELT |
| TNFRSF8 | TNFRSF4 |  | TMIGD2 |
| TNFRSF9 | TNFRSF8 |  | TNF |
| TNFSF10 | TNFRSF9 |  | TNFRSF10A |
| TNFSF11 | TNFSF13B |  | TNFRSF10B |
| TNFSF18 | TNFSF14 |  | TNFRSF10D |
| TNFSF8 | TNFSF8 |  | TNFRSF11A |
| VTCN1 |  |  | TNFRSF12A |
|  |  |  | TNFRSF13B |
|  |  |  | TNFRSF13C |
|  |  |  | TNFRSF14 |
|  |  |  | TNFRSF17 |
|  |  |  | TNFRSF18 |
|  |  |  | TNFRSF1B |
|  |  |  | TNFRSF21 |
|  |  |  | TNFRSF4 |
|  |  |  | TNFRSF8 |
|  |  |  | TNFRSF9 |
|  |  |  | TNFSF10 |
|  |  |  | TNFSF13 |
|  |  |  | TNFSF13B |
|  |  |  | TNFSF14 |
|  |  |  | TNFSF15 |
|  |  |  | TNFSF18 |
|  |  |  | TNFSF4 |
|  |  |  | TNFSF8 |
|  |  |  | VTCN1 |
